# Right Atrial Repair of Basal Ventricular Septal Rupture with Long-Term Follow-Up: A Case Report

**DOI:** 10.70352/scrj.cr.26-0450

**Published:** 2026-10-28

**Authors:** Masaru Yoshikai, Hisashi Sato, Nagi Hayashi

**Affiliations:** Department of Cardiovascular Surgery, Shin Koga Hospital, Kurume, Fukuoka, Japan

**Keywords:** basal ventricular septal rupture, right atrial approach, sandwich technique, tricuspid valve

## Abstract

**INTRODUCTION:**

Ventricular septal rupture (VSR) remains a life-threatening mechanical complication of acute myocardial infarction. Basal VSR is particularly challenging because of its deep location and close proximity to the atrioventricular valves. We report a case of successful repair of basal VSR through a right atrial approach using a double-patch sandwich technique.

**CASE PRESENTATION:**

An 88-year-old woman developed an 18-mm basal VSR following inferior myocardial infarction despite successful percutaneous coronary intervention. Mechanical circulatory support with an Impella CP (Abiomed, Danvers, MA, USA) was instituted; however, worsening pulmonary congestion and device-related hemolysis necessitated surgical intervention. Through a right atriotomy, the defect was directly exposed. After debridement of necrotic tissue, full-thickness sutures were placed through the ventricular septum, and the defect was closed using bovine pericardial patches on both the left and right ventricular aspects. Because the subvalvular apparatus of the septal leaflet was incorporated into the repair, concomitant tricuspid valve replacement was performed. Postoperative echocardiography demonstrated complete closure of the defect without residual shunt or deterioration of mitral valve function. The patient was discharged 7 weeks after surgery and remains free from recurrent heart failure, with no evidence of residual shunt at 5.5 years postoperatively.

**CONCLUSIONS:**

The right atrial approach combined with a double-patch sandwich technique provides excellent exposure of basal VSR and facilitates secure closure while avoiding ventriculotomy. This technique may represent a useful surgical option for selected patients with basal VSR.

## Abbreviations

IABP
: intra-aortic balloon pump
VSR
: ventricular septal rupture

## INTRODUCTION

VSR is one of the most devastating mechanical complications of acute myocardial infarction despite advances in reperfusion therapy. Surgical repair remains the standard treatment; however, operative mortality remains high because of tissue fragility, hemodynamic instability, and technical difficulties associated with closure of the defect. Basal VSR, as described by Cebeddu et al.^1)^ and classified according to the echocardiographic segmental classification,^2)^ is an uncommon subtype that presents unique surgical challenges because of its close relationship to the atrioventricular valves and subvalvular apparatus. Although several surgical strategies have been described, including infarct exclusion through left ventriculotomy^3)^ and patch repair through right ventriculotomy,^4)^ optimal surgical management of basal VSR remains controversial. We report a patient with postinfarction basal VSR who underwent successful repair through a right atrial approach^5)^ using a double-patch sandwich technique, with favorable long-term outcomes.

## CASE PRESENTATION

An 88-year-old woman was emergently admitted with chest pain and acute heart failure. She was diagnosed with an inferior myocardial infarction and underwent percutaneous coronary intervention for right coronary artery occlusion. Due to hemodynamic instability, an IABP was inserted. The IABP was removed on hospital day 8; however, she subsequently developed dyspnea. Transthoracic echocardiography revealed an 18-mm VSR in the basal ventricular septum (**Fig. 1**)^1,2)^ with mild mitral and tricuspid regurgitation. Cardiac catheterization demonstrated a pulmonary-to-systemic flow ratio of 6.2, indicating a significant left-to-right shunt. The pulmonary artery pressure was 60/28 mmHg (mean 43 mmHg), and the mean pulmonary artery wedge pressure was 28 mmHg. An Impella CP (Abiomed, Danvers, MA, USA) was inserted for circulatory support with the intention of stabilizing the patient and delaying surgical intervention. However, pulmonary congestion worsened, and hemolysis, presumably associated with the Impella device, progressed. Consequently, surgical intervention was performed on hospital day 11.

**Fig. 1:**
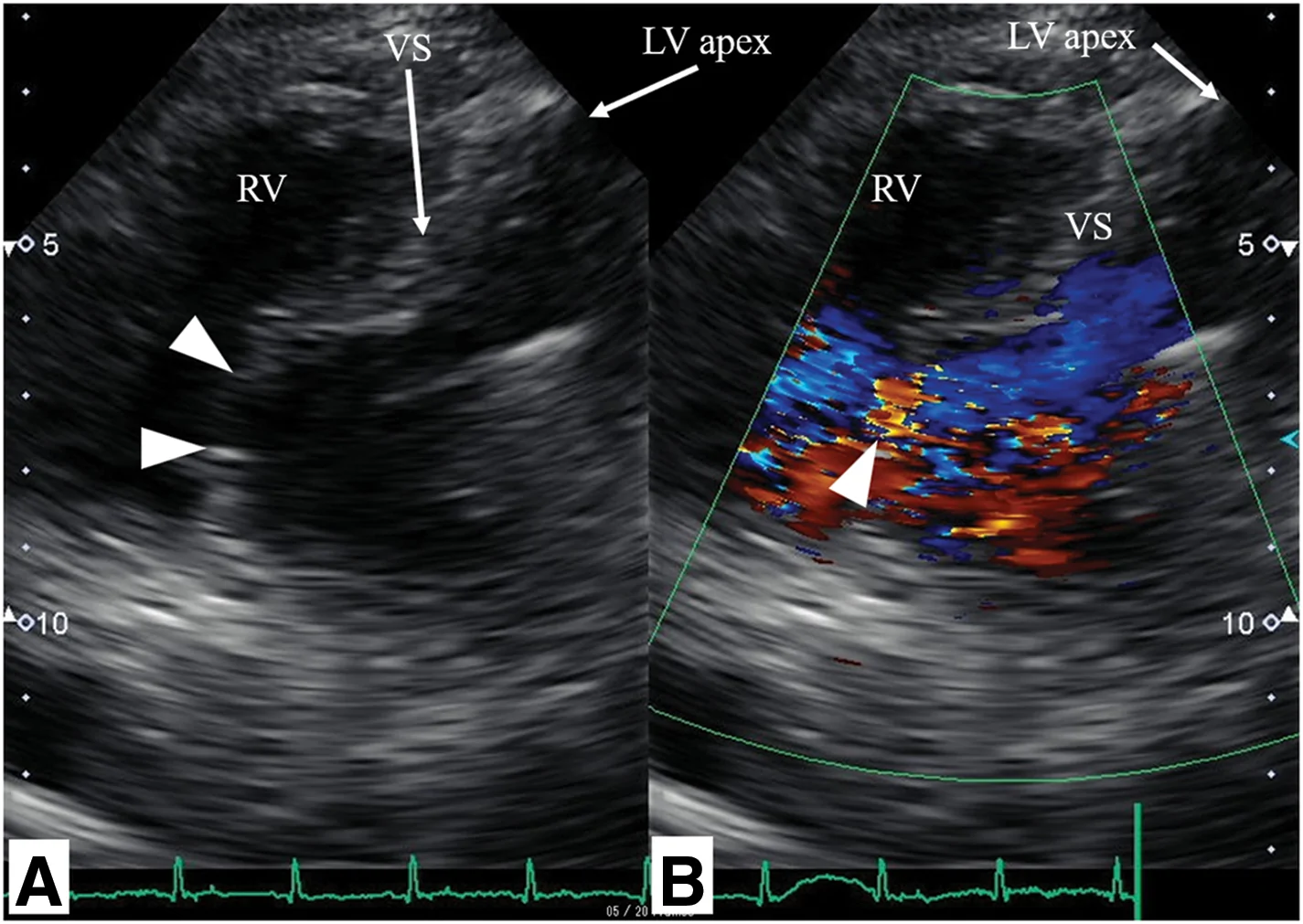
Transthoracic echocardiographic findings. The apical view showing a defect in the basal VS (arrowheads in **A**) and a left-to-right shunt jet through the defect from the LV to the RV (arrowhead in **B**). LV, left ventricle; RV, right ventricle; VS, ventricular septum

Intraoperative epicardial echocardiography demonstrated that the VSR was located immediately adjacent to the tricuspid annulus. The Impella CP was removed at the time of initiation of cardiopulmonary bypass. Systemic cooling was commenced, and ventricular fibrillation was induced, followed by a right atriotomy to identify the VSR. Cardiac arrest was then achieved. The VSR was found to run obliquely within the basal ventricular septum on the tricuspid annular side of the chordae supporting the septal leaflet of the tricuspid valve, approximately 1 cm from the tricuspid annulus (**Fig. 2A**). After division of these chordae (**Fig. 2B**), necrotic myocardium surrounding the VSR was debrided (**Fig. 2C**), which allowed clear visualization of the mitral valve complex. Full-thickness mattress sutures were first passed through the left ventricular-side bovine pericardial patch and then from the left ventricular aspect of the septum to the right ventricular side, traversing the healthy myocardium surrounding the defect (**Fig. 3A**). Particular attention was paid to directing the needle tip along the left ventricular surface of the septum to avoid entrapment of the mitral valve complex. The sutures were subsequently passed through the right ventricular-side patch and tied to secure both patches simultaneously (**Fig. 3B**), creating a double-patch sandwich configuration. No surgical adhesive or glue was used between the patches. Some of the suture exit points on the right ventricular side were located on the tricuspid annular side of the chordae supporting the septal leaflet (**Fig. 3A**). These sutures, together with division of the aforementioned chordae (**Fig. 2A** and **2B**), resulted in loss of functional support of the septal leaflet (**Fig. 3B**). Therefore, the septal leaflet was considered nonfunctional, and tricuspid valve replacement was performed using a bioprosthetic valve. The patient was successfully weaned from cardiopulmonary bypass with catecholamine support alone. Postoperative transthoracic echocardiography demonstrated complete closure of the defect without residual shunt. Mitral regurgitation did not worsen, and left ventricular function was preserved. The postoperative course was uneventful, and the patient was discharged 7 weeks after surgery, as postoperative recovery and rehabilitation were prolonged due to advanced age. At 5.5-year follow-up, she remained alive and free from recurrent heart failure with no evidence of residual shunt.

**Fig. 2:**
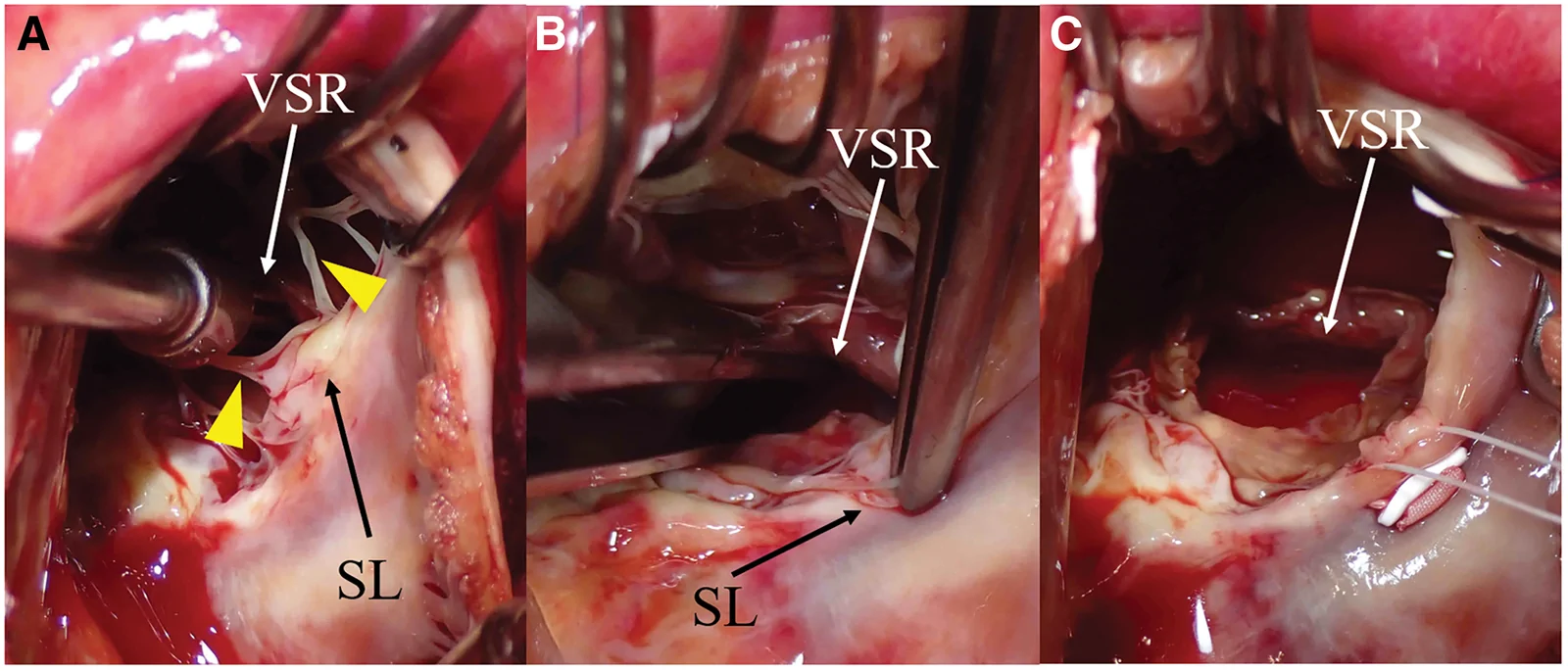
Operative findings show that the VSR is located on the tricuspid annular side of the chordae supporting the SL of the tricuspid valve (**A**). After division of these chordae, the obliquely coursing VSR becomes visible (**B**). Necrotic myocardium surrounding the VSR is then resected, and the VSR is enlarged (**C**). SL, septal leaflet; VSR, ventricular septal rupture

**Fig. 3:**
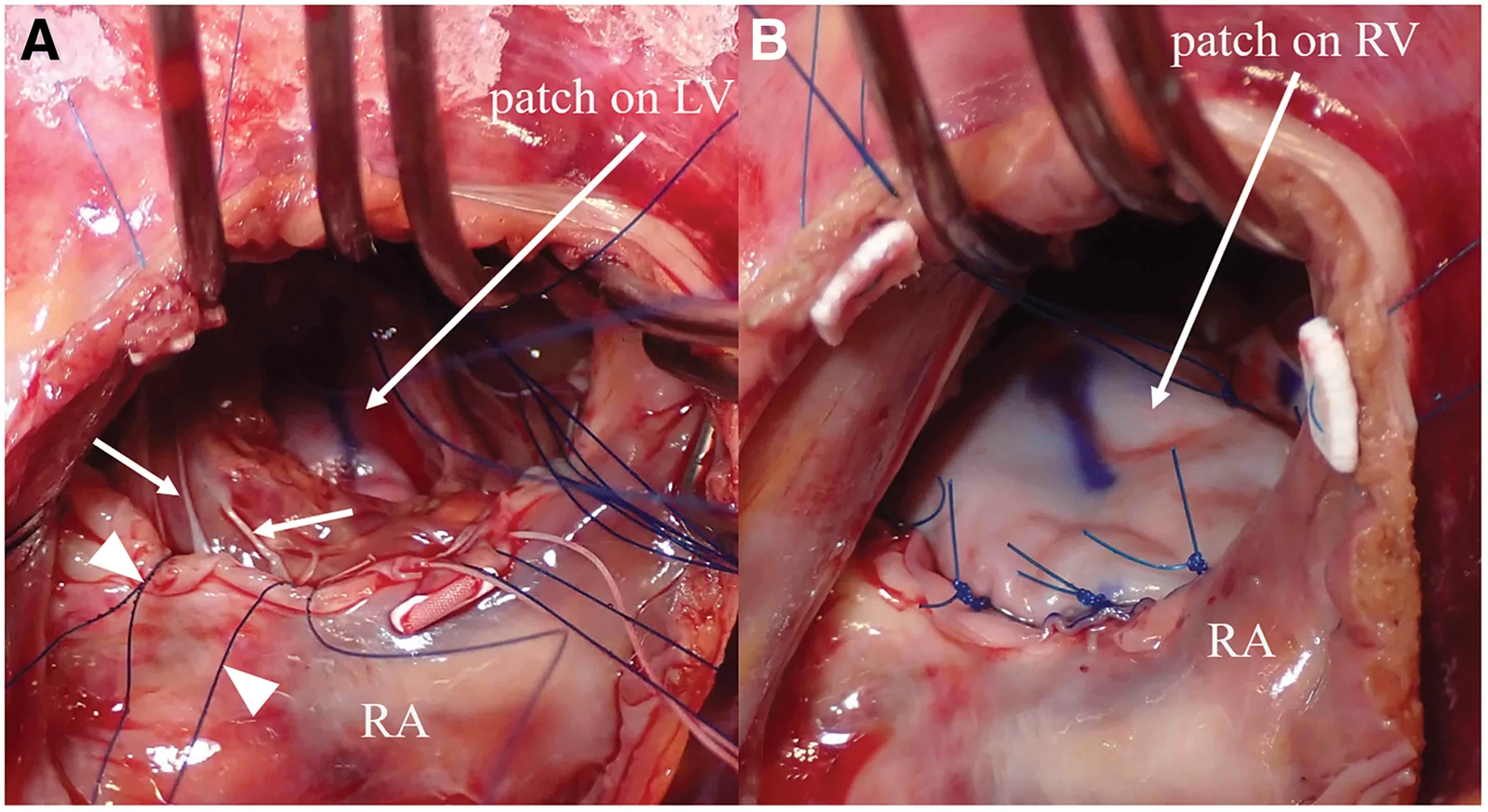
Operative findings. The right ventricular exit of sutures (arrowheads in **A**) transmurally placed through the ventricular septum is located on the tricuspid annular side of the origin of the chordae (arrows in **A**) supporting the septal leaflet of the tricuspid valve. Panel (**B**) shows completion of the double-patch sandwich technique. LV, left ventricle; RA, right atrium; RV, right ventricle

## DISCUSSION

Basal VSR is an uncommon form of postinfarction VSR. Unlike anterior VSR, basal VSR is located adjacent to the atrioventricular valves, making surgical exposure and secure patch placement technically demanding.^4)^ Although several surgical approaches have been reported, no consensus has been established regarding the optimal strategy for this subset of patients.

The anatomical characteristics of basal VSR contribute substantially to these technical challenges. The septal leaflet of the tricuspid valve is supported by multiple papillary muscles, many of which originate directly from the ventricular septum.^6)^ In addition, the presence of the muscular atrioventricular septum results in the tricuspid annulus being positioned more apically than the mitral annulus. Consequently, basal VSR is located adjacent to the tricuspid valve apparatus, and the papillary muscles supporting the septal leaflet frequently arise from the infarcted septal tissue surrounding the defect.

Left ventriculotomy with infarct exclusion is a widely used surgical approach for VSR.^3)^ However, in basal VSR, the defect is located deep within the ventricular base, resulting in a limited operative view. The mitral valve and the posteromedial papillary muscle further complicate the suture line, making accurate patch placement difficult and increasing the risk of residual shunt. In addition, left ventriculotomy carries inherent risks, including injury to the posteromedial papillary muscle, bleeding from the ventricular incision,^7)^ and postoperative arrhythmias.

The right ventricular approach with a double-patch sandwich technique is another established option for VSR repair and has the advantage of avoiding a left ventriculotomy.^4)^ This approach allows secure fixation of patches to both sides of the septum. However, in basal VSR, suture placement may be technically challenging because of the deep location of the defect and its proximity to the atrioventricular valve apparatus.^4)^ Thus, although the right ventricular approach is an effective alternative, it may not always provide optimal access for secure suture placement in basal VSR. Right ventriculotomy may also cause additional myocardial injury and may adversely affect right ventricular function or contribute to postoperative arrhythmias.

In contrast, the right atrial approach provides a direct and orthogonal view of the basal VSR without incision of either ventricle. Following debridement of necrotic myocardium around the VSR and/or separating tricuspid valve,^8)^ the mitral valve complex can be visualized directly through the defect. This facilitates accurate placement of sutures along the left ventricular aspect of the septum while minimizing the risk of incorporating the mitral valve apparatus into the patch repair. In the present patient, this exposure enabled secure closure using a double-patch sandwich technique without ventriculotomy, resulting in successful VSR closure without a residual shunt. In our opinion, among patients with basal VSR, those with a defect located close to the tricuspid annulus may be particularly suitable for repair through a right atrial approach.

However, this technique has an important implication for tricuspid valve function. The septal leaflet and its supporting structures are often incorporated into the right ventricular patch, resulting in loss of septal leaflet function. Experimental canine studies have suggested that removal of the septal leaflet does not result in significant tricuspid regurgitation in the acute phase.^9)^ However, the long-term consequences of isolated septal leaflet dysfunction remain unclear. Clinical experience with pacemaker lead–related tricuspid valve dysfunction suggests that mechanical impairment of the septal leaflet alone can result in tricuspid regurgitation, either through leaflet impingement or adhesion of the lead to the leaflet.^10)^ Once tricuspid regurgitation develops, progressive right ventricular dilatation and annular enlargement may further exacerbate regurgitation, potentially creating a vicious cycle. Therefore, although the available evidence is limited and the long-term consequences of septal leaflet dysfunction after VSR repair remain uncertain, leaving a non-functional septal leaflet *in situ* may carry a potential risk of late tricuspid regurgitation. In selected patients, concomitant tricuspid valve replacement may therefore be considered.^7,8)^

The favorable long-term outcome observed in this patient suggests that the right atrial approach combined with a double-patch sandwich technique can provide durable repair in selected patients with basal VSR.

## CONCLUSIONS

Repair of basal VSR through a right atrial approach using a double-patch sandwich technique provided excellent exposure of the defect and enabled secure closure without ventriculotomy. Although concomitant tricuspid valve surgery may be required, this strategy represents a useful option for selected patients with basal VSR and yielded satisfactory long-term results in the present case.

## DECLARATIONS

### Availability of data and materials

Data are available on request to the corresponding author.

## References

[1] Cubeddu RJ, Lorusso R, Ronco D, et al. Ventricular septal rupture after myocardial infarction: *JACC* Focus Seminar 3/5. J Am Coll Cardiol 2024; 83: 1886–901. 10.1016/j.jacc.2024.01.041 38719369

[2] Cerqueira MD, Weissman NJ, Dilsizian V, et al. Standardized myocardial segmentation and nomenclature for tomographic imaging of the heart: a statement for healthcare professionals from the Cardiac Imaging Committee of the Council on Clinical Cardiology of the American Heart Association. Circulation 2002; 105: 539–42. 10.1161/hc0402.102975 11815441

[3] David TE. Post-infarction ventricular septal rupture. Ann Cardiothorac Surg 2022; 11: 261–7. 10.21037/acs-2021-ami-111 PMC9207689 35733715

[4] Kinoshita T, Asai T, Hachiro K, et al. Extended sandwich patch technique via right ventriculotomy for acute ventricular septal rupture. Ann Thorac Surg 2022; 113: 1200–7. 10.1016/j.athoracsur.2021.04.090 34023237

[5] Massetti M, Babatasi G, Le Page O, et al. Postinfarction ventricular septal rupture: early repair through the right atrial approach. J Thorac Cardiovasc Surg 2000; 119: 784–9. 10.1016/S0022-5223(00)70014-6 10733770

[6] Dahou A, Levin D, Reisman M, et al. Anatomy and physiology of the tricuspid valve. JACC Cardiovasc Imaging 2019; 12: 458–68. 10.1016/j.jcmg.2018.07.032 30846121

[7] Sharma V, Greason KL, Nkomo VT, et al. Repair of acute inferior wall myocardial infarction–related basal ventricular septal defect: transatrial versus transventricular approach. J Card Surg 2013; 28: 475–80. 10.1111/jocs.12156 23870081

[8] Kitabayashi K, Miyake K, Sakagoshi N. Right atrial approach for repairing a posterior ventricular septal rupture: a case report. Surg Case Rep 2016; 2: 85. 10.1186/s40792-016-0215-9 PMC5005227 27576460

[9] Higashidate M, Tamiya K, Kurosawa H, et al. Role of the septal leaflet in tricuspid valve closure: consideration for treatment of complete atrioventricular canal. J Thorac Cardiovasc Surg 1992; 104: 1212–7. 1434697

[10] Alnaimat S, Doyle M, Krishnan K, et al. Worsening tricuspid regurgitation associated with permanent pacemaker and implantable cardioverter-defibrillator implantation: a systematic review and meta-analysis of more than 66,000 subjects. Heart Rhythm 2023; 20: 1491–501. 10.1016/j.hrthm.2023.07.064 37506990

